# Early autologous and allogeneic peripheral blood stem cell transplantation for adult patients with acute B and T cell precursor neoplasms: a 12-year single center experience

**DOI:** 10.1007/s00277-020-04391-x

**Published:** 2021-01-26

**Authors:** Normann Steiner, L. Brunelli, G. Hetzenauer, B. Lindner, G. Göbel, J. Rudzki, I. Peschel, M. Nevinny-Stickel, W. Nussbaumer, W. Mayer, L. Loacker, B. Kircher, E. Gunsilius, D. Wolf, D. Nachbaur

**Affiliations:** 1 University Hospital of Internal Medicine V, Hematology and Medical Oncology, Innsbruck Medical University, Innsbruck, Austria; 2 Department of Medical Statistics, Informatics and Health Economics, Innsbruck Medical University, Innsbruck, Austria; 3 Central Institute for Medical and Chemical Laboratory Diagnostics, Innsbruck Medical University, Innsbruck, Austria; 4 Department of Radiooncology, Innsbruck Medical University, Innsbruck, Austria; 5 Central Institute for Blood Transfusion and Immunology, Medical University Hospital Innsbruck, Innsbruck, Austria

**Keywords:** Acute lymphoblastic leukemia/lymphoma, Autologous, Allogeneic, Stem cell transplantation

## Abstract

Adult acute lymphoblastic leukemia/lymphoma (ALL/LBL) is a rare and heterogeneous malignancy characterized by uncontrolled proliferation of B or T cell precursor cells. Here, we retrospectively analyzed the outcome of early autologous stem cell transplantation in standard-risk patients in first complete remission (*n*=24) and of allogeneic transplantation in high and highest risk, and relapsed/refractory patients (*n*=35). The 10-year overall survival after autologous transplantation was 45%. The 10-year overall survival after allogeneic transplantation was 58%. The cumulative incidence of relapse was 29% after allogeneic and 67% after autologous transplantation. The cumulative incidence of non-relapse mortality was 0% after autologous and 12% after allogeneic transplantation. This retrospective single center analysis in a limited number of standard-risk patients clearly demonstrates that early autologous transplantation in first complete remission leads to an acceptable long-term outcome with a short overall treatment duration of less than 6 months compared with more than 2 years with conventional chemotherapy. More sensitive and standardized methods to detect minimal residual disease (MRD) will further help to identify those patients more accurately who are most likely to benefit from such a short and intensive treatment strategy (i.e., MRD negative standard-risk patients) or those who require early targeted therapy (e.g., blinatumomab) in case of MRD positivity. Early allogeneic transplantation results in long-term survival/cure in nearly two-thirds of all high and highest risk, and relapsed/refractory patients.

## Introduction

Acute lymphoblastic leukemia/lymphoma (ALL/LBL) is a rare and heterogeneous hematological malignancy. Uncontrolled proliferation of lymphoid progenitor cells committed to the B or T cell lineage in the bone marrow, the peripheral blood, and/or lymphatic and extra-lymphatic tissue characterize the disease. The estimated age- and disease-dependent annual incidence in Europe ranges between 0.17 and 1.45 per 100,000 individuals [1]. Over the past decades, treatment results have improved due to optimized risk stratification, the implementation of monoclonal antibodies (rituximab), BiTE antibodies (blinatumomab), and antibody-drug conjugates (inotuzumab-ozogamicin), as well as the development of highly sensitive diagnostic tools for MRD assessment [2–6]**.** Besides patient- and disease-related risk-factors and response dynamics, MRD has evolved to the most sensitive marker for the risk of relapse after conventional chemotherapy as well as after stem cell transplantation (SCT) [1, 4, 7]. Allogeneic stem cell transplantation (SCT) is the treatment of choice for high and highest risk as well as relapsed/refractory patients [2, 3]. The conventional treatment approach for standard-risk patients consists of remission induction, consolidation, intensification, and maintenance with an overall treatment duration of 2 or more years [4]. The regimens most commonly used for adults in Europe are based on the pediatric BFM (Berlin-Frankfurt-Münster) protocol, whereas in the USA and many other parts of the world, the hyper-CVAD regimen is preferred [1, 8]. Despite such intensive treatment algorithms, results for adult ALL patients remain unsatisfying [4]. The role of autologous SCT (ASCT) in order to improve treatment results remains controversial. Clinical trials including randomized studies in the pre-MRD era in unselected ALL cohorts have failed to demonstrate any beneficial effect of such a treatment approach [2, 5]. However, studies that are more recent have shown that ASCT might be a suitable option at least for specific subgroups [6, 7].

The main goal of our retrospective single center analysis was to assess whether a significant reduction of treatment duration by early consolidation with ASCT in first complete remission in adult standard-risk ALL is feasible without loss of efficacy compared with the usually recommended conventional post-remission consolidation/maintenance strategy with an overall treatment duration of 2.5–3 years.

## Patients and methods

### Patients

Between March 2008 and October 2019, fifty-nine adult patients with newly diagnosed B- or T-precursor ALL/LBL received either an autologous (*n*=24) or allogeneic (*n*=35) SCT. All patients gave written informed consent. Virtually all patients including those with T-LBL received remission induction (Phase I and II) and consolidation I according to the German Multicenter ALL Protocol for Adults (GMALL 07/2003 Amendment 6, 30.06.2010; Consensus Guidelines of the German Multicenter Study Group for the Treatment of T-lymphoblastic Lymphoma in Adults, Version 1, 18.01.2011) or in patients > 55 years of age according to the GMALL Elderly Protocol 1/2003 (Amendment 5, 21.06.10). Patients with Philadelphia chromosome positive (Ph+) ALL additionally received imatinib 600 mg daily according to the respective protocol.

Characteristics of patients receiving a first ASCT are listed in Table 1. All patients with B- or T-precursor ALL had standard-risk disease as defined by the GMALL 07/2003 protocol. Autologous peripheral blood stem cells were mobilized with granulocyte-colony stimulating factor (G-CSF) 10–20 μg/day starting on day 10 after the first consolidation cycle according to the GMALL 07/2003 protocol. All stem cell products were tested leukemia-free based on conventional fluorescence-activated cell sorter (FACS) analysis, conventional cytogenetics including fluorescence in-situ hybridization (FISH) analysis, and Ig/TCR clonality GeneScan analysis. Median time from diagnosis to transplant in patients in first complete remission (CR1) was 4.9 (range, 2.9–8.9) months. Only one patient received the transplant 20.2 months after diagnosis in second complete remission (CR2) because of a life-threatening infection during consolidation I. Complete remission was determined by bone marrow examination including cytology, conventional FACS analysis, conventional cytogenetics, FISH analysis, real-time polymerase-chain reaction (RT-PCR), Ig/TCR clonality GeneScan analysis, and/or positron emission tomography/computed tomography (PET/CT) scan and/or cerebrospinal fluid assessment when indicated. Additionally, since 2019 next-generation sequencing (NGS) and since April 2019 MRD diagnostics by next-generation flow cytometry (NGF), according to EuroFlow consensus, recommendations (sensitivity < 10^−5^) were available. According to The GMALL recommendations for stem cell transplantation, all patients received a conditioning regimen with fractionated total body irradiation (fTBI, 12 Gray) combined with either cyclophosphamide (120 mg/m^2^) or etoposide (60 mg/kg). One patient refusing total body irradiation received intravenous busulfan (12.8 mg/kg) and melphalan (140 mg/m^2^). All patients with T-LBL (*n*=7) received the BEAM (carmustine, etoposide, cytarabine, melphalan) regimen [9, 10].

**Table 1:** Characteristics of standard-risk patients receiving an autologous stem cell transplantation in first or second complete remission (*n*=24)

Median age at the time of diagnosis (years, range)	28 (18–66)
Male/female ratio	16:8
Median time from diagnosis to ASCT in patients in CR1 (months, range)	4.9 (2.9–8.9)
ALL subclassification	
B precursor, Ph*−*	14
T precursor	3
T-LBL	7
Remission status at the time of ASCT	
CR1	23
CR2	1
Conditioning regimen	
TBI-containing (12 gray)	16
BUMEL	1
BEAM	7
Median CD34 cell number (×10^6^/kg BW, range)	3.74 (1.17–20.87)
Median number of days to leukocyte engraftment (≥ 1.0 G/L)	11 (9–14)

*Abbreviations*: *ALL*, acute lymphoblastic leukemia; *Ph−*, Philadelphia chromosome negative; *LBL*, lymphoblastic lymphoma; *CR1*, first complete remission; *CR2*, second complete remission; *TBI*, total body irradiation; *BUMEL*, busulfan, melphalan; *BEAM*, carmustine, etoposide, cytarabine, melphalan; *BW*, body weight

Characteristics of patients receiving a first allogeneic SCT are listed in Table 2. All patients receiving an allogeneic SCT in CR1 had high- or highest-risk disease as defined by the GMALL 07/2003 protocol and received remission induction and consolidation according to this protocol (*n*=29, 83%). One patient was in CR2, and five patients had relapsed/refractory disease. Median time from diagnosis to SCT was 4.5 (range, 1.2–14.8) months. The majority of the patients (83%) had precursor B cell ALL with seven patients being Philadelphia chromosome positive (Ph1+). Conditioning was TBI-containing (fTBI, 12 Gray) and myeloablative in 22/35 (63%) patients, and of fludarabine-based reduced intensity with or without lower doses of fractionated TBI (fTBI, 8 Gray) in the remaining 13/35 (37%) patients. Graft-versus-host disease prophylaxis was calcineurin inhibitor based combined with either methotrexate in the myeloablative setting (*n*=22) or with mycophenolate mofetil in the reduced-intensity setting (*n*=13). Eighteen patients (51%) received a graft (mainly peripheral blood stem cells) from an HLA-identical sibling donor. Twelve patients (34%) received allogeneic transplants from HLA-matched unrelated donors (10/10). Five patients (14%) receiving a graft from HLA-mismatched unrelated donors additionally received anti-thymocyte globulin (Grafalon®, 30–60 mg/kg) prior to transplant. All but one patient who died on day+1 because of septic shock engrafted after a median of 13 (range, 8–21) days.

**Table 2:** Characteristics of high- and highest-risk and relapsed/refractory patients receiving an allogeneic stem cell transplantation (*n*=35)

Median age at the time of diagnosis (years, range)	44 (20–70)
Male/female ratio	13:22
Median time from diagnosis to SCT (months, range)	4.5 (1.2–14.8)
ALL subclassification	
B precursor, Ph*−*	22
B precursor, Ph+	7
T precursor	6
Remission status at the time of SCT	
CR1	29
CR2	1
R/R	5
Conditioning regimen	
Myeloablative TBI-containing (12 gray)	22
Reduced-intensity	
TBI-containing (8 Gray)	4
Chemotherapy only	9
Stem cell donor	
HLA-ID sibling	18
Matched unrelated (10/10)	12
1-Ag mismatched unrelated	5
Median donor age (years, range)	33 (13–66)
Stem cell source	
BM	2
PB	33
Recipient/donor sex combination	
m/f	7
Others	28
Median CD34 cell number (×10^6^/kg BW, range)	6.15 (2.91–18.9)
Median number of days to leukocyte engraftment (≥ 1.0 G/L)	13 (8–21)

*Abbreviations*: *SCT*, stem cell transplantation; *ALL*, acute lymphoblastic leukemia; *Ph−*, Philadelphia chromosome negative; *Ph+*, Philadelphia chromosome positive; *CR*, complete remission; *R/R*, relapsed/refractory; *TBI*, total body irradiation; *HLA-ID*, human leukocyte antigen-identical; *AG*, antigen; *BM*, bone marrow; *PB*, peripheral blood; *m/f*, male/female; *BW*, body weight

Thirteen patients in the autologous group relapsed after a median of 8.2 (range, 2.8–77.1) months, with only two patients having late relapses > 2 years after ASCT. Twelve patients proceeded to an allogeneic SCT within a median of 1.9 (1.3–3.2) months. All but one patient received fludarabine-, clofarabine-, or nelarabine-based salvage therapy prior to transplant [11, 12]. Only four patients achieved a CR2 or CR3. All other patients (8/12, 67%) had refractory disease at the time of allogeneic SCT. Conditioning was of reduced intensity in all patients (fludarabine/busulfan/melphalan (FBM), *n*=8; thiotepa/busulfan/fludarabine, *n*=4). All but two patients who died too early due to septic multi-organ failure engrafted after a median of 13 (range 8–23) days.

### Study endpoints

The primary endpoint of this retrospective single center analysis was overall survival (OS). Secondary endpoints were disease-free survival (DFS), non-relapse mortality (NRM), and relapse incidence (RI).

### Statistical methods

Data were retrospectively reviewed and analyzed as of February 2020. All statistics were computed using NCSS Statistical Software version 19.0.5. The probabilities of OS were calculated using the Kaplan-Meier method from the date of the first transplant until death. DFS was calculated from the date of the first transplant until relapse or death whichever occurred first. The cumulative RI was calculated from the date of the first transplant until relapse with death without relapse as competing risk. The cumulative incidence of NRM was calculated from the date of the first transplant to the date of death without prior relapse with death from relapse as competing risk. NRM for patients receiving an allogeneic SCT because of relapse after a first ASCT was calculated until the date of the allogeneic SCT [13].

## Results

### Autologous peripheral blood stem cell transplantation in adult standard-risk patients with B- and T-precursor ALL/LBL in first complete remission

**Fig. 1:**
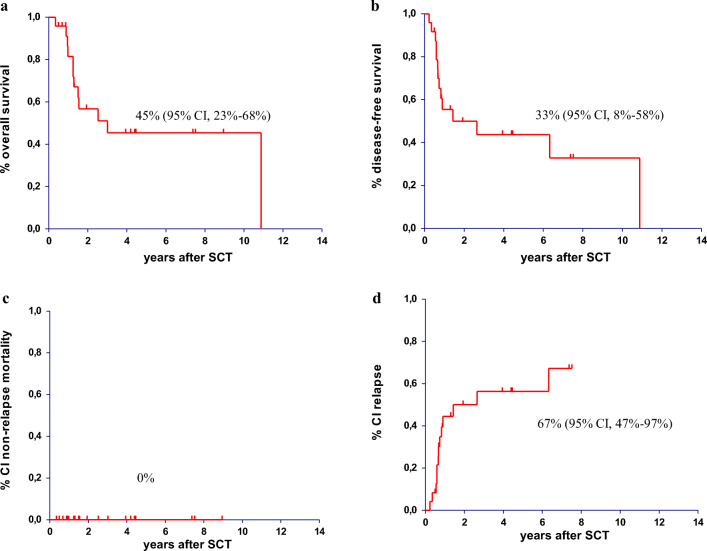
Autologous stem cell transplantation in adult standard-risk patients with B- and T-precursor ALL/LBL in first complete remission. **a**) overall survival **b**) disease-free survival **c**) cumulative incidence of non-relapse mortality **d**) cumulative incidence of relapse

The 10-year OS and DFS for the entire cohort was 45% (95% CI, 23–68%) and 33% (95% CI, 8–58%), respectively (Fig. 1a and b). One patient died a natural death more than 10 years after ASCT in ongoing complete remission. The NRM after ASCT was 0% (Fig. 1c). Thirteen patients relapsed with the original leukemic clone with two patients having late relapses > 2 years after ASCT. The resulting cumulative RI at 5 and 8 years after ASCT was 56% (95% CI, 38–83%) and 67% (95% CI, 47–97%), respectively (Fig. 1d).

### Allogeneic stem cell transplantation in high- and highest-risk patients in first complete remission or relapsed/refractory B- and T-precursor ALL/LBL

**Fig. 2:**
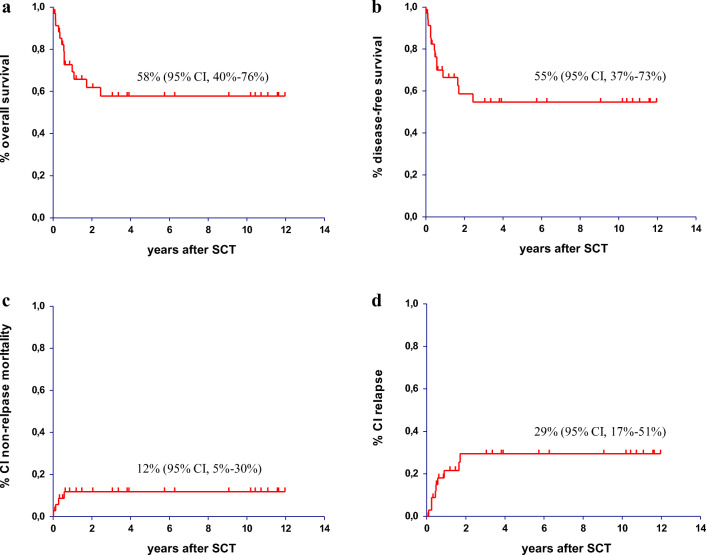
Allogeneic stem cell transplantation in high-and highest-risk patients in first complete remission or relapsed/refractory B- and T-precursor ALL/LBL. **a**) overall survival **b**) disease-free survival **c**) cumulative incidence of non-relapse mortality **d**) cumulative incidence of relapse

The 10-year OS for the entire cohort was 58% (95% CI, 40–76%) (Fig. 2a). The 10-year DFS was 55% (95% CI, 37–73%) (Fig. 2b). The 2- and 5-year NRM was 12% (95% CI, 5–30%) (Fig. 2c). This apparently low NRM most likely resulted from the relatively high proportion of reduced-intensity conditioning used for the transplants (37%). Although NRM was higher in the myeloablative setting (15%; 95% CI, 5–42%) compared with reduced-intensity conditioning (8%; 95% CI, 1–50%), this difference was statistically not significant due to low patient number. Nine patients relapsed within the first 2 years after transplant and the cumulative RI was 30% (95% CI, 17–53%) (Fig. 2d). No relapse occurred > 2 years from transplant.

Twelve (34%) patients developed acute graft-versus-host disease ≥ grade II resulting in a significantly better 5- and 10-year OS (92% vs 42%, *p =* 0.02) and DFS (79% vs 42%, *p* = 0.05) mainly due to a trend towards a lower RI compared with patients without acute graft-versus-host disease (13% vs 38%, *p* = 0.07). There was no difference in NRM between patients with or without acute graft-versus-host disease ≥ grade II (data not shown).

Chronic graft-versus-host disease occurred in 7/32 (22%) patients at risk surviving > 100 days after transplant. There were trends towards a better OS (71% vs 55%, *p* = 0.33) and a better DFS (71% vs 50%, *p* = 0.26) due to a lower RI (0% vs 39%, *p* = 0.06), with no difference in NRM (14% vs 11%) for patients with chronic graft-versus-host disease compared with patients without chronic graft-versus-host disease.

Moreover, no significant differences in outcome and RI of patients with T cell precursor or Ph+ ALL compared with other B-precursor subtypes were observed after allogeneic SCT (data not shown).

### Allogeneic stem cell transplantation for relapse after ASCT

The outcome of patients receiving an allogeneic SCT for relapse after ASCT (*n*=12) was dismal with only two patients surviving > 2 years (OS 11%; 95% CI, 0–38%). It is of note that the majority of patients (8/12; 67%) were refractory to either fludarabine-, clofarabine-, or nelarabine-based salvage regimen prior to allogeneic SCT. As expected, this poor outcome was mainly due to a high 1-year RI of 50% (95% CI, 28–88%) and a high 1-year NRM of 33% (95% CI, 15–74%).

## Discussion

This retrospective single center analysis demonstrates that early intensification with ASCT in CR1 in adult patients with standard-risk B- or T-precursor ALL/LBL achieves a long-term OS and DFS of 45% and 33%, respectively, without any treatment-related mortality. Although heterogeneous due to the retrospective nature of our analysis, the distribution of the immunologic subtypes corresponds to that observed within the GMALL studies, and all patients including those with T-LBL received the same induction and intensive consolidation according to the GMALL 07/2003 protocol and the GMALL consensus statement for the treatment of adult patients with T-LBL. Despite the short overall treatment duration with a median time from diagnosis to transplant of 4.9 months, our survival data are comparable to the results of the LALA-94 trial in which standard-risk patients receiving remission induction, consolidation, and a consecutive maintenance program for 2 years achieved a 5-year OS and DFS of 44% and 35%, respectively [14]. In the MRC UKALLXII/ECOG E2993, multinational trial patients were randomized by a donor versus no donor stratification after remission induction and 3 courses of high-dose methotrexate to either receive an allogeneic transplant or, if no donor was available, to receive either consolidation/maintenance or an autograft. Among those patients randomized to chemotherapy versus autograft, the 5-year OS in standard-risk patients was 46% with chemotherapy and only 37% with an autograft [5]. Better results were reported by the PETHEMA ALL-AR-03 trial, in which only high-risk patients were eligible. Patients with good early cytological response and MRD negativity at the end of consolidation were allocated to delayed consolidation/maintenance therapy instead of allogeneic SCT. The 5-year OS and DFS of these MRD negative high-risk patients was 58% and 52%, respectively, particularly emphasizing the importance of highly sensitive MRD measurements for treatment guidance [15].

Whether MRD negativity determined by highly sensitive tools can also be used to significantly reduce the overall treatment duration by early intensification with ASCT in standard-risk patients in CR1 remains an open question. Besides the limited patient number, the fact that NGF for highly sensitive MRD detection was not available in our institution before April 2019 is another major drawback of our retrospective single center analysis [16]. Minimal residual disease negativity by NGF was therefore only documented in 1/1 patient in the autogroup and in 4/5 patients in the allogroup after this diagnostic tool has been implemented. However, although no firm conclusion can be drawn for such small patient numbers, we are convinced that the implementation of NGF will further improve the outcome of our early autotransplant strategy especially by reducing the risk of relapse through an optimized selection of those standard-risk patients who are most likely to benefit from such a short and intensive treatment approach, i.e., MRD negative patients. Furthermore, standard-risk patients who do not achieve MRD negativity (MolCR as defined by ESMO Guidelines) are candidates for one or two early courses of blinatumomab [1]. With blinatumomab, MRD negativity can be achieved in another 70–80% of MRD positive patients as has been demonstrated by the BLAST trial [17]. Whether in blinatumomab responders a consolidation with an autologous transplant with low or even no mortality, as reported here, would be preferable over allogeneic transplantation at least in standard-risk patients because of the high NRM of 37% seen in the BLAST Trial remains an open question [18]. Moreover, it remains elusive whether successful stem cell mobilization is feasible after the administration of blinatumomab to enable subsequent autotransplant at all [19].

Relapse of the underlying disease was the sole reason for the failure of our concept of a short and intensive treatment duration with a final autotransplant. The majority of these relapses were refractory to standard of care salvage chemotherapy thereby resulting in a poor outcome even after subsequent allogeneic stem cell transplantation. Our data in this patient cohort is comparable with the recently published report from the EBMT Acute Leukemia Working Party on the results of allogeneic stem cell transplantation with sequential conditioning using the FLAMSA-RIC strategy in adult patients with refractory or relapsed acute lymphoblastic leukemia with an overall survival of only 17% and a graft-versus-host disease- and leukemia-free survival of only 14% at 2 years [20]. Better results are only obtainable when MRD negativity prior to allotransplant is achieved by implementing targeted therapies [21, 22]. Whether a subsequent allogeneic SCT can be omitted in MRD responders after blinatumomab in the relapsed/refractory setting as it has been supposed by the results of the phase 3 TOWER study has to be interpreted with caution [22].

While the use of ASCT in adult ALL remains a matter of debate, frontline allogeneic SCT is the treatment of choice for Ph1+ and other high-risk B- and T-precursor neoplasms [23–25]. Our results in thirty-five high- and highest-risk and relapsed/refractory patients perfectly fit to the recently published retrospective data of the Acute Leukemia Working Party of the European Society for Blood and Marrow Transplantation (EBMT) showing a long-term survival/cure in about two-thirds of the patients, a relatively low NRM of < 20%, and a RI of about 30% [26]. Whether in the era of potent tyrosine kinase inhibitors (TKIs) in combination with BiTE antibodies or antibody-drug conjugates allogeneic SCT can be replaced by ASCT with less morbidity and mortality at least in those high- and highest-risk patients achieving a deep MolCR remains to be shown by prospective randomized trials.

In conclusion, despite all limitations, our retrospective single center analysis clearly demonstrates that an early autotransplant strategy significantly shortens treatment duration while providing an acceptable long-term outcome in standard-risk patients in first complete remission. The implementation of NGF might be helpful to better define those patients who would benefit most from such a short and intensive treatment approach by reducing the risk of relapse and the consecutive need for a salvage allotransplant with dismal outcome. The increasing availability of MRD evaluation by standardized, highly sensitive diagnostic tools and potent-targeted therapies (e.g., blinatumomab, TKIs) not only allows a more individualized medicine in adult ALL but also asks for the need to reconsider and redefine the role of prolonged chemotherapy versus early autologous or allogeneic stem cell transplantation in the treatment algorithms of various ALL subgroups.

## Data Availability

The datasets generated or analyzed during this study are available from the corresponding author on reasonable request.
